# Rare manifestations of alobar holoprosencephaly and the potential causes: a report of two cases

**DOI:** 10.1097/MS9.0000000000000176

**Published:** 2023-02-07

**Authors:** Wael Nakawa, Sandy Alkhalil, Nafiza Martini, Ieman Alawad

**Affiliations:** a Damascus University; b Stemosis for Scientific Research, Damascus; c Alassad Medical Complex, Hama, Syrian Arab Republic

**Keywords:** alobar holoprosencephaly, anencephaly, case report, cebocephaly, cyclopia, rachischisis

## Abstract

Holoprosencephaly is a rare and possibly fatal neural tube defect represented by complete or partial forebrain noncleavage. It can be classified into four types: alobar, semilobar, lobar, and middle interhemispheric fusion variant. It is usually diagnosed through prenatal ultrasound or after birth by visually observing the morphological abnormalities and/or through neurological screening. Potential causes include maternal diabetes, alcoholism, infections during pregnancy, drugs, and genetic causes.

**Case presentation:**

Herein, we report two cases of holoprosencephaly’s rarest manifestations, albeit cebocephaly in the first case, and cyclopia with a probocis in the second. Cebocephaly, (hypotelorism with a single nostril and a blind-ended nose) was present in the first case; a Syrian newborn girl for a 41-year-old mother who works in collecting *Capparis spinosa*, and cyclopia with skull vault absence and posterior encephalocele in the second case; a Syrian newborn girl for a 26-year-old mother, the parents here where second-degree relatives.

**Conclusions:**

Early diagnosis through ultrasound is preferred in such cases and management options should be assessed and discussed with the parents due to poor prognosis. Adherence to pregnancy follow-up programs is essential to detect malformations and disorders as early as possible, especially when risk factors exist. Also, this paper may suggest a potential correlation between *C. spinosa* and holoprosencephaly. Therefore, we suggest that more research should be done.

HIGHLIGHTS

- Alobar holoprosencephaly (HPE) is a rare and possibly fatal neural tube defect represented by complete or partial forebrain noncleavage.
- It can be classified into four types: alobar, semilobar, lobar, and middle interhemispheric fusion variant.
- Early diagnosis through ultrasound is preferred in such cases and management options should be assessed and discussed with the parents due to poor prognosis.
- This paper may suggest a potential correlation between *Capparis spinosa* and HPE. Therefore, we suggest that more research should be done.

## Introduction

HPE is the most frequent and complex human brain malformation during the development of the forebrain. It is the result of impaired or incomplete division of the forebrain (prosencephalon), which should have divided into right and left hemispheres^1^,^2^. Studies showed that it occurs between the 18th and the 28th day of gestation^1^,^3^. Being the most common forebrain developmental anomaly, it has a prevalence estimated by 1/16 000 live borns, and an incidence as high as 1:250 in conceptuses^3^. Most severe cases of HPE are often first identified on prenatal ultrasound, others are frequently diagnosed during the neonatal period through facial abnormalities and/or neurological presentation, which initiates further screening^1^. HPE is generally categorized into four types based on the degree of nonseparation. These types include alobar HPE which is the rarest, and most clinically severe, it involves no separation of the cerebral hemispheres and a single brain ventricle ‘monoventricle.’ The semilobar form is characterized by the fusion of the left and right frontal and parietal lobes, while posterior lobes are spared as the interhemispheric fissure exists posteriorly. The lobar form, in which the frontal lobes are fused, especially ventrally, while most of the right and left cerebral hemispheres and lateral ventricles are spared. Another type of HPE is called ‘The middle interhemispheric variant.’ In this type, posterior, frontal, and parietal lobes fail to separate, a varying lack of cleavage of thalami and the basal ganglia and an absent body of the corpus callosum, while more polar parts of the cerebrum are fully separated and the splenium and the genu of the corpus callosum are present^1^,^2^. The severity of craniofacial malformations and prognosis tend correlate to the degree of nonseparation: the alobar form is the most severe in terms of both craniofacial malformations and neurological impairment^2^.

In this type of HPE, there is a limited formation of the anterior portion of the brain. It also entails a lack of interhemispheric fissure, a falx cerebri, or a corpus callosum. There is no third ventricle and the thalami are fused. The partially formed cerebrum is found in the rostral calvarium. A dorsal cyst is almost always found in alobar HPE (92%), which correlates with thalamic fusion. Craniofacial findings may also be present. These may include ocular hypotelorism (decreased distance between eyes), cyclopia (a single, midline, fused eye that exists in a single orbit below a proboscis), proboscis (a nose-like appendage), ethmocephaly (proboscis separating ocular hypotelorism), cebocephaly (ocular hypotelorism with a single nostril nose), single nostril, or cleft lip and/or palate^4^.

The work has been reported in line with the SCARE 2020 criteria^5^.

## Case presentation

### Case 1

A 41-year-old multigravida (G6P5) came to the maternity unit at 33 weeks of gestation complaining of uterine contractions. Her medical and surgical history was unremarkable. Consanguineous marriage and familial malformations and smoking were all denied. She negated any drug exposure or infection during pregnancy. She had no antenatal checkups or ultrasounds done because she lives in an area affected by the war in Syria. She also mentioned that she worked in picking ‘*Capparis spinosa*’ during her pregnancy. She had four healthy children and one dead infant because of cerebral atrophy; as she claimed. Laboratory tests were within normal limits. An ultrasound on admission showed severe polyhydramnios and hydrocephalus.

**Figure 1:**
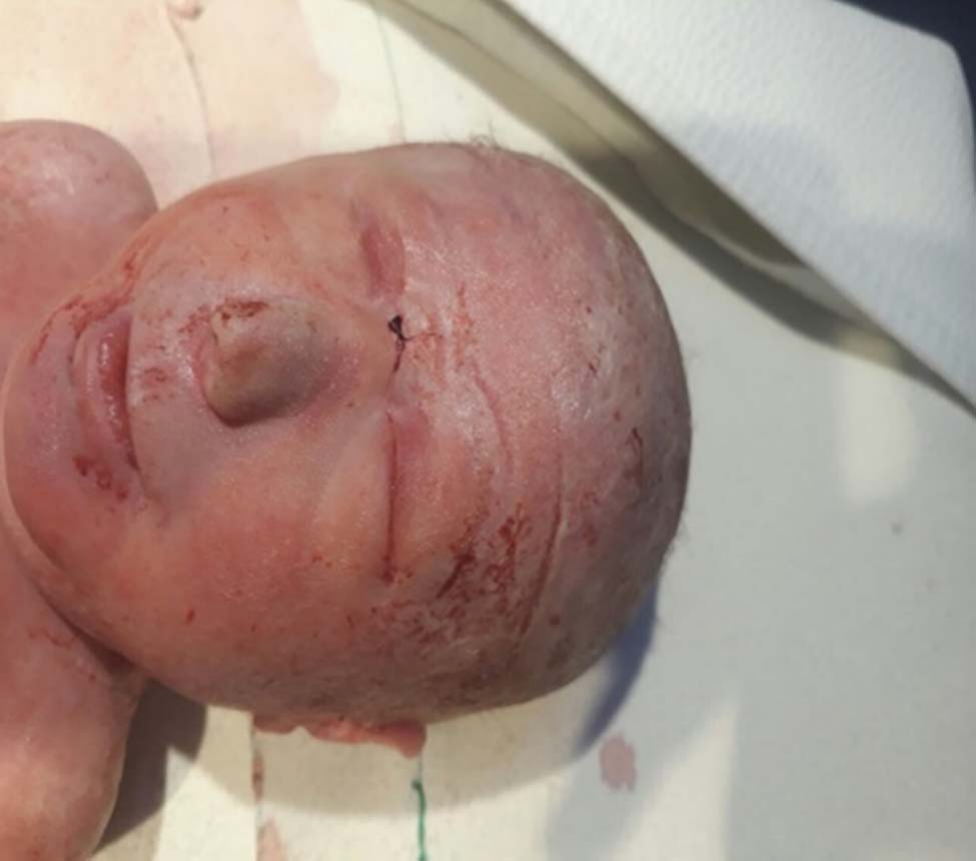
Special facial features including hypotelorism with a single nostril.

**Figure 2:**
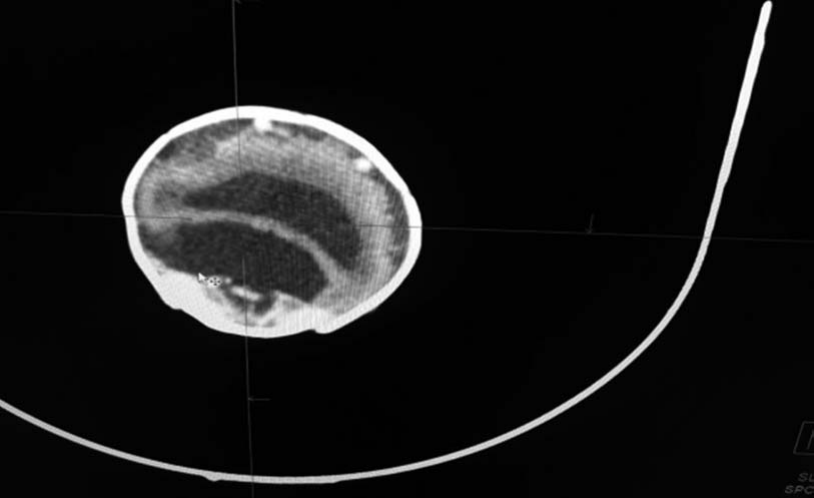
Computed tomography scan shows a single, severely dilated ventricle in the midline of the brain. Cisterna magna is very dilated. There are no clear anatomical landmarks of corpus callosum, thalamus or falx cerebri.

**Figure 3:**
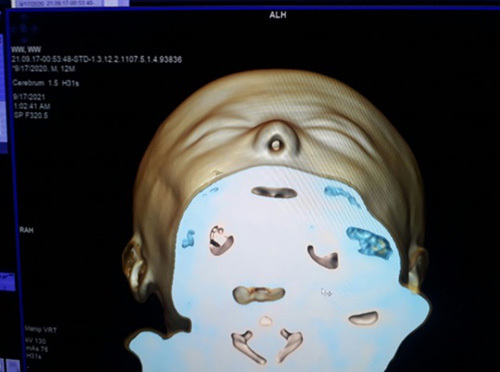
A single nasal cavity with a single nostril.

**Figure 4:**
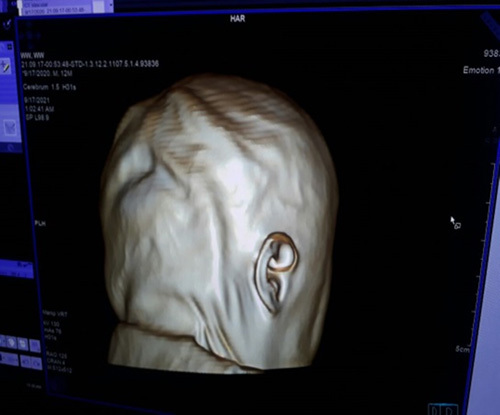
Craniosynostosis.

A spontaneous development of labor occurred with spontaneous rupture of membranes followed by normal vaginal delivery of an alive female newborn. The newborn died 2 hours after delivery after several resuscitation tries. On examination, special facial and cranial features were noted, including hypotelorism with a single nostril, a blind-ended nose, and craniosynostosis (Fig. 1). Other organs were normal. The esophagus was patent as it enabled passage of a nasogastric tube that was passed through the mouth. Computed tomography scan showed a single, severely dilated ventricle in the midline of the brain characterized in alobar HPE. Cisterna magna was also very dilated. There were no clear anatomical landmarks of a corpus callosum, a thalamus, or a falx cerebri, a single nasal cavity that ends blindly, and a single nostril were also observed (Figs. 2–4). Therefore, the diagnosis of cobocephaly was done. Fortunately, the mother was discharged the next day as she did not have any complications.

The parents refused any fetal autopsy or chromosomal studies.

### Case 2

A 26-year-old multigravida (G2P1) came to the maternity unit complaining of sticky liquid vaginal leakage with regular uterine contractions. She is a nonsmoker and nonalcoholic with no significant medical or surgical history. She was followed during her pregnancy in a special clinic through checkups and ultrasounds which at the time showed an absent skull vault, a severe neural tube deformation and an incomplete spinal fusion in cervical vertebrae that reaches the lumbar area, and alobar HPE with anencephaly was initially diagnosed. The mother and her husband (who is her first cousin) were advised to the need for termination of pregnancy, however, they denied the termination.

At admission, blood examinations were normal. An ultrasound before the delivery revealed an absent skull vault and polyhydramnios.

**Figure 5:**
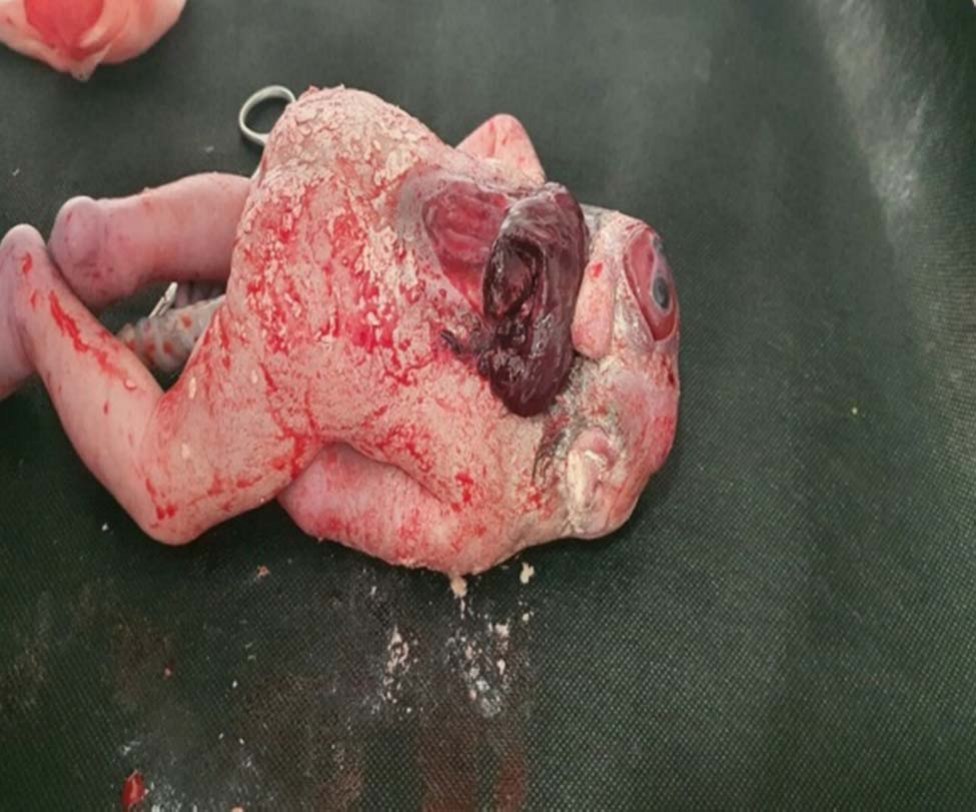
The Skull vault is absent with posterior encephalocele and open spinal cord.

**Figure 6:**
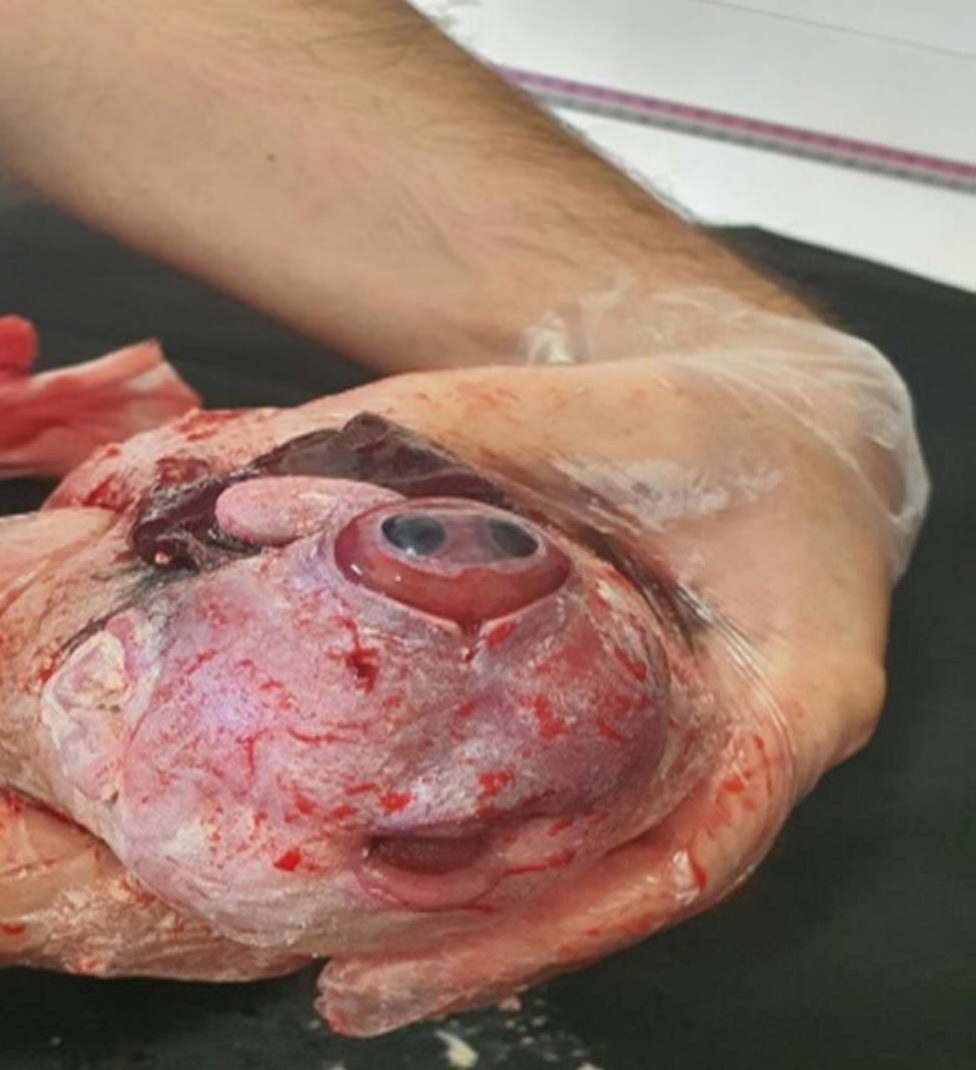
A proboscis measuring 22 mm in length and 10 mm in diameter is present at the superior aspect of the face. A single median orbit with a well-developed globe partially covered by eyelids present in between the mouth and proboscis.

A spontaneous delivery of a female stillbirth occurred. It had the following findings with gross examination: a proboscis measuring 22 mm in length and 10 mm in diameter which was present at the superior aspect of the face, a single median orbit with a well-developed globe partially covered by eyelids was present in between the mouth and proboscis, the Skull vault was absent with rachischisis that reaches the lumbar vertebrae, the nose was absent. The ears, mouth, palate, and tongue were normal, so were the hands, feet, and genitalia (Figs. 5 and 6). Fortunately, the mother was also discharged the next day as she did not have any complications.

The diagnosis of alobar HPE associated with cyclopia with probocis and rachischisis was confirmed. Fetal autopsy was not possible due to refusal by the parents.

## Clinical discussion

Alobar HPE is considered the most severe type of HPE. It is characterized by the nonseparation of the cerebral hemispheres and a single brain ventricle ‘monoventricle.’ The severity of the facial defects correlates to the severity of cerebral defects in 80% of cases^3^. Considering that in our cases, both of them are on the severe end of HPE, showing two of the most severe facial deformities, albeit cebocephaly in the first case, and cyclopia with rachischisis in the second.

HPE has a heterogeneous etiology, potential causes include maternal diabetes, alcoholism, infections during pregnancy, drugs such as retinoic acid, chromosomal syndromes such as trisomy 13, and recessive autosomal mutations like SHH, SIX3, and ZIC2. It can also happen sporadically in isolated cases^3^. Overall, 50% of all HPE cases have trisomy 13, while 70% of cases diagnosed with trisomy 13 have HPE^3^,^4^.

Trisomy 13 which is named ‘Patau’s syndrome’ cannot be emphasized as a cause of these two cases since karyotype was not available in either case due to lack of means during war time. The mother in the first case worked in collecting capers flowers before and during her pregnancy. *C. spinosa* is used in traditional medicine since ancient times and it is alleged to show antioxidant and antibacterial effects^6^. Studies on the effects of this medicinal plant during pregnancy are close to nonexistent, one study by Davari *et al.* 7 suggested the possibility of teratogenic potential of *C. spinosa* after conducting their study on pregnant mice. We theorize the effect of *C. spinosa* on neural tube formation and development, and we suggest for more research to be done on the subject.

The second case had second-degree consanguinity, where the parents were first cousins. Consanguinity is shown to be associated with a higher risk of congenital malformations and recessive autosomal diseases^8^. HPE, has four types, middle interhemispheric, lobar, semilobar, and alobar, with alobar being the rarest and most severe type. It manifests in midline facial abnormalities, such as cyclopia, ethmocephaly, cebocephaly, and premaxillary agenesis^9^. Cebocephaly, which is characterized by hypotelorism and a single nostril that ends blindly^10^, was present in our first case where attempts to insert a nasogastric tube were futile. Craniosynostosis and hypotelorism were also present.

Cyclopia is the most severe manifestation of alobar HPE. It is a fatal defect with an estimated prevalence of one in 100 000 births, including stillbirths^11^. Rachischisis is also a severe and fatal neural tube defect characterized by anencephaly accompanied by a bony defect of the spine and exposure to neural tissue^12^. Anencephaly is the absence of structures derived from the forebrain and skull where the forebrain and midbrain are absent or replaced by rudimentary fibrovascular tissue with scattered islands of neural elements^13^.

Cyclopia with proboscis, anencephaly, and rachischisis were all present in our second case making it (to the best of our knowledge) the 11th documented case of such severe and extremely rare association^12^. Children with alobar HPE have a poor prognosis. They are usually stillbirths or die shortly after birth^1^. Our first case died after 2 hours of birth, while the second was a stillbirth.

Early diagnosis through antenatal ultrasound is recommended to assess the case and explore management possibilities. However, that was not possible in our first case due to the parents being in a war-affected area, and although our second case was diagnosed through prenatal ultrasound, we could not provide the ultrasounds as the ultrasound machine could not print them due to technical difficulties.

Furthermore, unlike our cases, termination of pregnancy is advised in such severe cases^10^. The parents in our first case could not reach medical assistance during the pregnancy, while the parents in the second case refused abortion.

## Conclusion

Alobar HPE is a rare, possibly fatal developmental defect of the embryonic forebrain with heterogeneous etiology. Early diagnosis through ultrasound is preferred and management options for such cases should be assessed and discussed with the parents due to poor prognosis. Fetal autopsy and chromosomal studies may help in determining its specific etiology.

This paper may suggest, to a certain extent, a correlation between *C. spinosa* and HPE. Therefore, we recommend for more research should be done to determine the effects of *C. spinosa* during pregnancy.

## Ethics approval and consent to participate

Not applicable.

## Consent for publication

Written informed consent was obtained from the patient for publication of this case report and accompanying images. A copy of the written consent is available for review by the Editor-in-Chief of this journal on request.

## Sources of funding

Not applicable.

## Authors’ contributions

W.N. and S.A. contributed to drafting, reviewing, and bibliography. N.M. contributed to drafting, reviewing, corresponding, data collecting, and bibliography. E.A. contributed to drafting, data collecting, supervising, reviewing, and editing. All authors read and approved the final manuscript.

## Conflicts of interest disclosure

The authors declare that they have no financial conflict of interest with regard to the content of this report.

## Research registration unique identifying number (UIN)

Not applicable.

## Guarantor

Eiman Alawad.

## Provenance and peer review

Not commissioned, externally peer reviewed.
